# Effects of La Addition on the Microstructure, Thermal Conductivity and Mechanical Properties of Mg-3Al-0.3Mn Alloys

**DOI:** 10.3390/ma15031078

**Published:** 2022-01-29

**Authors:** Huafeng Liu, Jing Zuo, Taiki Nakata, Chao Xu, Guisong Wang, Hailong Shi, Guangze Tang, Xiaojun Wang, Shigeharu Kamado, Lin Geng

**Affiliations:** 1 School of Materials Science and Engineering, Harbin Institute of Technology, Harbin 150001, China; 20B909019@stu.hit.edu.cn (H.L.); zuo_jing@126.com (J.Z.); wangguisong@hit.edu.cn (G.W.); xjwang@hit.edu.cn (X.W.); genglin@hit.edu.cn (L.G.); 2 Department of Mechanical Engineering, Nagaoka University of Technology, 1603-1, Kamitomioka, Nagaoka 940-2188, Japan; nakata@mech.nagaokaut.ac.jp (T.N.); kamado@mech.nagaokaut.ac.jp (S.K.)

**Keywords:** Mg-Al-La-Mn alloys, texture, microstructure, thermal conductivity, mechanical properties

## Abstract

The effects of La addition on the microstructure, thermal conductivity and mechanical properties of as-cast and as-extruded Mg-3Al-xLa-0.3Mn (x = 1, 3 and 5 wt.%) alloys were studied. The results showed that the thermal conductivity of the alloys increased with the addition of La element, which was due to the formation of the Al_11_La_3_ phases by consuming the solute Al and the added La element. The yield strength of the as-cast Mg-3Al-xLa-0.3Mn alloys increased with the increase in La concentration. The thermal conductivity of the as-extruded alloys was lower than that of as-cast counterparts owing to lots of defects generated in the process of hot extrusion deformation, particularly the grain boundaries. The anisotropy of thermal conductivity was discovered in the as-extruded alloys on account of the formation of texture. As the La content increases, the tensile strength and yield strength of the as-extruded alloys decreased gradually. In contrast, the elongation first increased and then decreased, resulting from the combined effect of the texture strengthening and second phase strengthening.

## 1. Introduction

Magnesium (Mg) alloys have wide application potential in the 3C products aerospace, automobile industries owing to their lightweight, superior specific strength, excellent casting performance and high thermal conductivity [1,2,3,4,5]. As the integrated circuits advance by leaps and bounds, higher demands have been brought up for the performance of materials, including high strength, low density and great heat dissipation performance. Mg alloys with high thermal conductivity enable the rapid transformation of heat. Consequently, the reliability and service life of the electronic devices are greatly improved because performance deterioration caused by over-heat could be prevented as the node temperature of the electronic devices could be cooled effectively [6].

The thermal conductivity of pure Mg is as high as 156 W/(m·K) [3]. However, the mechanical properties of pure Mg are too poor, which restrict its practical application [7]. Alloying is an efficient method to ameliorate the mechanical properties of Mg alloys. For example, aluminium (Al) is one of the most widely used alloying elements in Mg alloys, which can effectively improve the strength, hardness and casting properties [8,9,10]. However, the Al addition could distort the lattice of the Mg matrix, then enhances the electrons/phonons scattering and reduce the mean free path of the electrons/phonons [7], and hence reducing the thermal conductivity of the Mg-Al alloys [6,11,12,13]. Furthermore, the Mg_17_Al_12_ phase is the major strengthening phase in Mg-Al alloys, which has poor thermal stability at high temperatures. Thus, the dissolution of Mg_17_Al_12_ phase increases the Al content in the Mg matrix and results in the deterioration of the thermal conductivity [3].

It was reported that the affixion of rare-earth (RE) elements to the Mg-Al alloys gives rise to the Al-RE intermetallic compounds that possess high melting points and good thermal stability, which can enhance the tensile properties and creep resistance at elevated temperatures of the Mg alloys [14,15,16]. Besides, the Al-RE intermetallic compounds deplete Al solutes that dissolve in the Mg matrix, which makes the alloy maintain high thermal conductivity. However, the variation in the RE elements has an apparent impact upon the mechanical properties and thermal conductivity of the Mg-Al based alloys [17,18,19,20,21,22]. The solid solubility of light rare earth elements like La, Ce and Nd is low in the Mg matrix so the RE addition mainly contributes to strengthening by grain refinement. Among them, La has lower solid solubility in the Mg matrix, which was supposed to be better for the maintenance of the high thermal conductivity [23]. The adjunction of La consumes the Al solute in Mg-Al alloy to form the intermetallic compound Al_11_La_3_, which has fine lamellar morphology and excellent thermal stability. These Al_11_La_3_ phases can increase the strength and high-temperature performance of the alloys [24,25]. Hence, numerous studies concentrate on the impact of La on the casting properties, mechanical properties and creep resistance of Mg-Al based alloys [14,15,16,24,25,26,27]. However, the effect of La on the thermophysical properties, especially the thermal conductivity of the Mg-Al based alloys, was seldom reported, and the mechanism of action is not thoroughly analyzed.

In the present work, the microstructure, mechanical properties and thermal conductivity of the Mg-3Al-xLa based alloys were systematically studied. The impact of the La concentration on the microstructure evolution, mechanical properties and thermal conductivity of Mg-3Al-xLa-0.3Mn alloys were discussed, which provided a reference for the research and development of high thermal conductivity and high strength Mg alloys.

## 2. Experimental Procedures

Alloys with the composition of Mg-3Al-xLa-0.3Mn (x = 1, 3 and 5 wt.%) were prepared by permanent mould direct-chill casting, and the mass ratio of the chill mold to the casting is about 3:1. Pure Mg (99.89 wt.%), Mg-Al (30 wt.%), Mg-Mn (3 wt.%), Mg-La (30 wt.%) master alloys were melted and held at 760 °C in a mild steel crucible under the CO_2_/SF_6_ atmosphere, followed by water cooling. The chemical compositions (Table 1) of the ingots were identified utilizing an inductively coupled plasma mass spectrometer (iCAP 7400) (Thermo Fisher Scientific, Waltham, MA, USA). The cast ingots were processed into cylindrical samples with approximately 42 mm in diameter and 25 mm in height. The rods were indirectly extruded at 350 °C with the ram of 0.1 mm/s, followed by water cooling. The extrusion ratio was about 18:1 and the rods were preheated 15 min at 350 °C before extrusion.

The microstructural characterizations were conducted by scanning electron microscopy (SEM) equipped with energy dispersive spectrometer (EDS) and electron backscattered diffraction (EBSD) facility, and transmission electron microscope (TEM). The samples for the SEM observations were mechanically ground, polished and the SEM images were obtained in the secondary electron (SE) mode. The software Image-Pro Plus 6.0 was utilized to statistically estimate the phase region information of the microstructure image. The samples analyzed by EBSD were first ground with sandpaper, and then electropolished in the solution (90 vol.% ethanol + 10 vol.% perchloric acid) with the 0.2 A current value at a temperature below −20 °C for 20–30 s. The foils for TEM observation were prepared by mechanically polished to a thickness of about 50 µm and then ion-milled using Gatan Plasma Ion Polisher (GATAN682) (Gatan, Inc., Warrendale, PA, USA). The phase analyses were conducted by the X-ray diffractometer (XRD) with Cu K_α_ radiation.

Tensile tests were performed using AG-X Plus Shimadzu electronic universal testing machine (Shimadzu Company, Kyoto, Japan) at room temperature (RT) at a strain rate of 1 mm/min. The tensile specimens were machined from the cast ingots, and the extruded bars parallel to the extrusion direction (ED). The cross-sectional areas are 6 mm × 2 mm (as-cast samples) and 3 mm × 1.5 mm (as-extruded samples), the gauge length is 15 mm. The test samples of this experiment were obtained by Numerical Control Wire Cut EDM (Jiangsu Fangzheng CNC Machine Tool Co., LTD, Wuxi, China) on a wire cutting machine tool.

The disk samples (6 mm in diameter and 2 mm in thickness) for thermal diffusivity measurements were processed from the alloys. Samples of the as-extruded alloys were cut along and perpendicular to the ED, which was designated as ∥ED and ⊥ED, respectively. The thermal diffusivity (α) was measured on Netzsch LFA 447 device (NETZSCH Scientific Instruments Trading Ltd., Selb, German) by laser flash method at the temperature of 25 °C and at least three shots were operated. Before the test, a layer of graphite was sprayed uniformly on the upper and lower surfaces of the sample to enhance the absorption rate of the light pulse [28]. The specific heat capacity ($C_{P}$) at constant pressure in this study was obtained according to the Neumann–Kopp rule [29,30]. The density (*ρ*) was obtained by the Archimedes method. The thermal conductivity (λ) was calculated via the following equation:

(1) $$\lambda =\alpha \cdot \rho \cdot C_{P}$$

where *α* (mm^2^/s) is the thermal diffusivity, *ρ* (g/cm^3^) is the density at room temperature, $C_{P}$ (J/(g·K)) is the specific heat capacity.

## 3. Results and Discussion

### 3.1. Microstructure of Mg-3Al-xLa-0.3Mn Alloys

**Figure 1:**
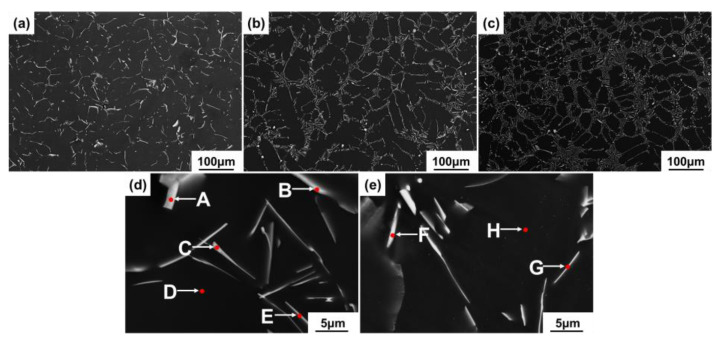
SEM-SE microstructures of as-cast Mg-3Al-xLa-0.3Mn alloys: (**a**) x = 1; (**b**) x = 3; (**c**) x = 5;(**d**,**e**) EDS analysis of the as-cast Mg-3Al-xLa-0.3Mn alloys, x = 3.

**Figure 2:**
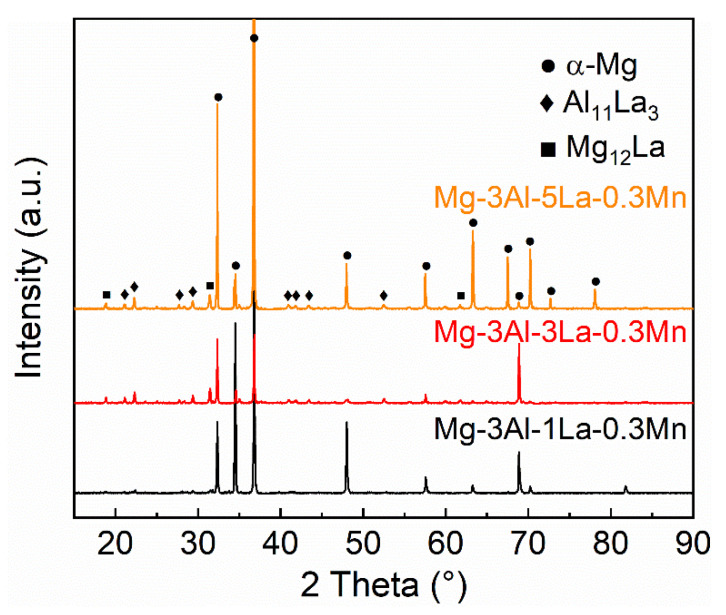
X-ray diffraction patterns of as-cast Mg-3Al-xLa-0.3Mn alloys.

Figure 1 shows the microstructure of the as-cast Mg-Al-La-Mn alloys, which are mainly composed of α-Mg matrix and the second phases distributed along the dendrite. The phases exhibit lamellar, rod-like and granular morphologies. The eutectic phase in the Mg-3Al-1La-0.3Mn alloy is intermittently distributed along the grain boundaries, and a small amount of granular second phase is dispersed in the grains. With increasing La content, the area fraction of the second phase in the alloys gradually increases, which are 5.9 vol.%, 10.8 vol.% and 14.7 vol.% as the amount of La varies from 1 wt.% to 3 wt.% and then to 5 wt.%. Besides, the semi-continuous network structure gradually transforms into a continuous network structure. The XRD spectrums of the as-cast alloys are given in Figure 2, which suggests that all the Mg-Al-La-Mn alloys are composed of α-Mg matrix, Al_11_La_3_ phases, and Mg_12_La phases. The compositions of the phases in the Mg-3Al-3La-0.3Mn alloy were analyzed utilizing SEM-EDS, as shown in Figure 1d,e and Table 2, which indicates that the needle-shaped second phase should be corresponding to the Al_11_La_3_ phases, while the granular and bulk shaped phases are determined to be Al_8_Mn_5_ and Mg_12_La phases. In reference [30], the contents of Mg and La in Al_8_Mn_5_ phase of AlLa42 and AlLa46 alloys are 21.87 at.%, 3.21 at.% and 17.02 at.%, 9.51 at.%, respectively. Furthermore, in this study, the atomic ratio of Al and Mn in the particle of Figure 1d is 33.91:16.51, which is closer to 8:5, so the phase is identified as Al_8_Mn_5_ phase. However, due to its low area faction, the Al_8_Mn_5_ phase cannot be detected in the XRD spectrum. The size of dendrite cells decreases with the La content increasing. The partition coefficient of the solute La is less than 1, and the solute atoms La and Al are enriched in the liquid before the solid-liquid interface during solidification. This may bring about the composition undercooling and the decrease of the rate of atom diffusion, thereby increasing the number of nuclei and limiting grain growth. Furthermore, the concentration of solute atoms results in the generation of Al-La phases that mainly distribute in the region of the grain boundary. Therefore, the grain growth is further restrained [14].

**Figure 3:**
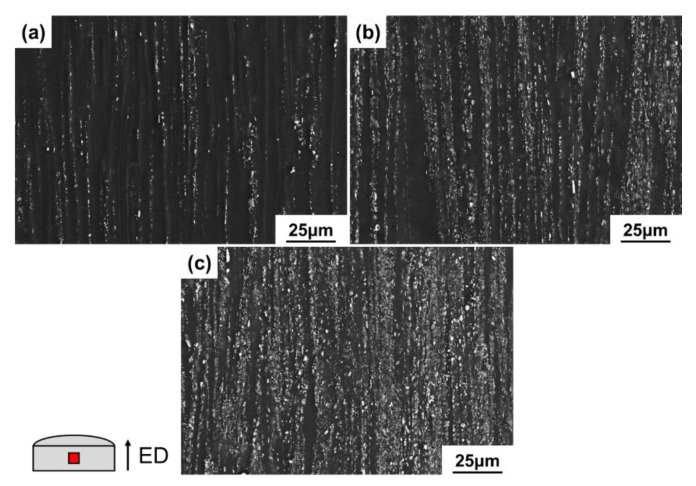
SEM-SE microstructures of as-extruded Mg-3Al-xLa-0.3Mn alloys: (**a**) x = 1; (**b**) x = 3; (**c**) x = 5.

Figure 3 shows the SEM micrographs of the extruded alloys observed perpendicular to the ED. The needle-shaped Al_11_La_3_ phases were fragmented into fine particles and distributed densely along the ED. On the other hand, the Al_8_Mn_5_ phases were relatively difficult to be fractured during the extrusion process and randomly distributed in the α-Mg matrix. As the amount of La increases, the volume fraction of the second phase gradually increases, as the La content are 1 wt.%, 3 wt.% and 5 wt.%, the corresponding volume fractions are 6.1 vol.%, 12.3 vol.%, and 17.1 vol.%, respectively. The volume fraction of second phase of the extruded alloys has increased in relation to the as-cast alloys, which may be caused by dynamic precipitation during extrusion.

**Figure 4:**
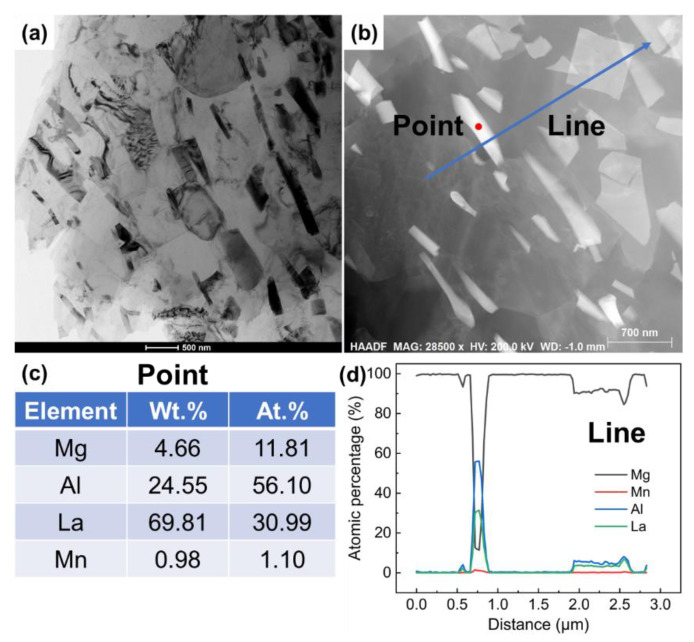
TEM observations of as-extruded Mg-3Al-5La-0.3Mn alloy: (**a**) TEM bright-field image; (**b**) HADDF image and EDS point/line analyses obtained from the as-extruded Mg-3Al-5La-0.3Mn sample; (**c**,**d**) EDS analysis results of the phase marked Point and Line in (**b**).

**Figure 5:**
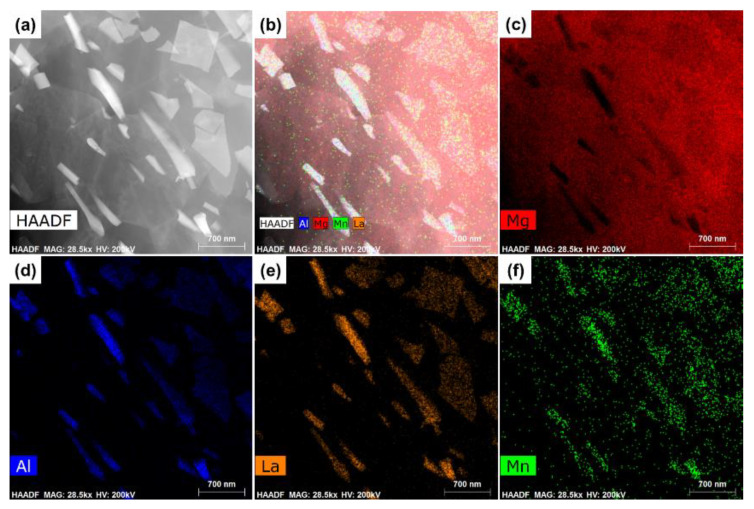
TEM microstructure of as-extruded Mg-3Al-5La-0.3Mn alloys (**a**) HAADF image; (**b**) EDS map analyses obtained from the as-extruded Mg-3Al-5La-0.3Mn sample; (**c**–**f**) elemental mapping of Mg, Al, La and Mn, respectively.

Figure 4 shows the TEM bright-field and high-angle annular dark-field (HAADF) images of the as-extruded Mg-3Al-5La-0.3Mn alloy, which confirm that the needle-shaped phases were fractured into sub-micron pieces. According to the EDS analyses, they should be Al_11_La_3_ phases. Figure 5 shows the elemental mapping of the same region given in Figure 4b. As expected, the matrix phase overwhelmingly contained Mg. Al, La and Mn were densely enriched at the areas of plate-like and irregular block second phase particles.

**Figure 6:**
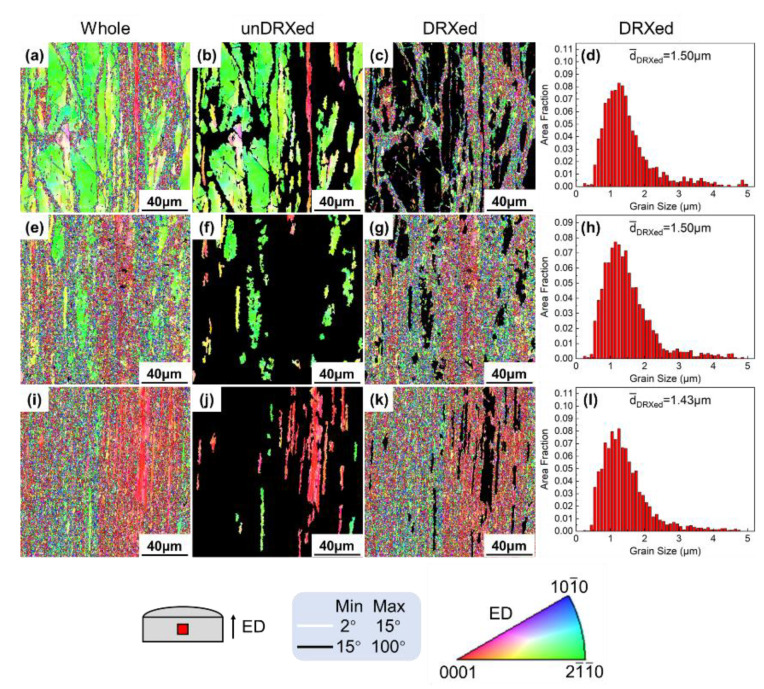
IPF maps of as-extruded Mg-3Al-xLa-0.3Mn alloys: (**a**–**c**) x = 1; (**e**–**g**) x = 3; (**i**–**k**) x = 5; grain size distribution histograms of DRXed region of the samples: (**d**) x = 1; (**h**) x = 3; (**l**) x = 5.

As shown in Figure 1, the majority of the second phases in the as-cast Mg-Al-La-Mn alloys are much larger than 1 μm, which can promote the dynamical recrystallization (DRX) during the extrusion process by particle stimulated nucleation (PSN) mechanism [31,32,33]. During the hot extrusion process, these coarse, brittle and thermal stable Al_11_La_3_ phases cannot be deformed and were broken into smaller particles. The dislocations in the Mg matrix prefer to accumulate near the Al_11_La_3_ phases, which provided nucleation sites for the dynamical recrystallization grains, thereby promoting the DRX process of the alloys [34]. As the amount of La increases, the area fraction of the second phase gradually increases, which provides more nucleation sites for the dynamic recrystallization during the hot extrusion, then enhances the DRX. Therefore, as shown in Figure 6, the proportion of dynamical recrystallization gradually increased with the increase of La content, and its values are 49.4%, 88.5%, and 89.2%, respectively. The finer second phase (<1 μm) (broken second phase and precipitation phase) has a pinning effect on the grain boundary, which can effectively impede the grain boundary migration and inhibit the growth of recrystallized grains, thereby refining the microstructure. Therefore, the DRXed grain sizes of extruded alloys with different La contents are very small and have little difference. The mean DRXed grain sizes of samples Mg-3Al-1La-0.3Mn, Mg-3Al-3La-0.3Mn and Mg-3Al-5La-0.3Mn are 1.50 µm, 1.50 µm and 1.43 µm, respectively.

In addition, in the process of extrusion deformation, a great deal of coarse second-phase particles have a strong hindrance to the slip of dislocations, and it will also cause insufficient dislocations to accumulate in some grains to drive the occurrence of dynamic recrystallization. As the amount of La increases, the content of the finer precipitated phase also gradually increases, which mainly plays a role in spiking grain boundaries rather than PSN of dynamic recrystallization, thereby hindering the nucleation of recrystallization and the diffusion of grain boundaries. It partially offsets the amount of PSN nucleation caused by the coarse second phase particles. Therefore, in contrast with the Mg-3Al-3La-0.3Mn alloy, although the content of the second phase increases, the proportion of dynamic recrystallization increases very little.

The DRXed grains show comparatively random orientations, while the deformed grains exhibit obvious basal plane orientation characteristics. The (0001) basal plane is parallel to ED, which means that the c-axis of the grains rotates during extrusion, and the c-axis is perpendicular to the ED after rotation. Obvious color contrast changes could be observed near the grain boundaries of the deformed grains, indicating that the orientation of the deformed grains near the grain boundaries is different, and there are sub-crystals and lots of dislocations. The number of deformed grains is relatively small, and the average grain size is large, which are 24.8 µm, 10.7 µm and 14.7 µm, respectively.

**Figure 7:**
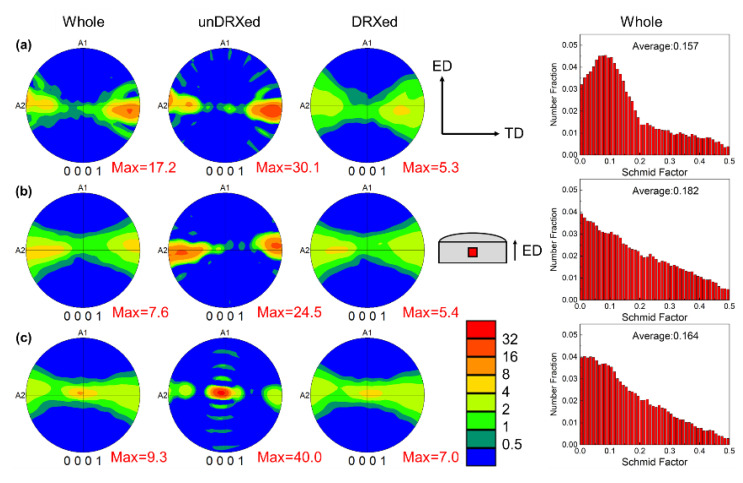
(0001) pole figures and Schmid factor distributions of as-extruded Mg-3Al-xLa-0.3Mn alloys: (**a**) x = 1; (**b**) x = 3; (**c**) x = 5.

Figure 7 shows the (0001) pole figure and Schmid factor distribution diagram of $\{0001\}\langle 11\overset{-}{2}0\rangle$ basal slip system of the as-extruded Mg-3Al-xLa-0.3Mn alloys with different La content. The as-extruded samples show the typical basal fiber texture and the basal planes parallel to the ED. As shown in Figure 7, the intensity of basal texture of the DRXed region in the as-extruded alloy is much weaker than that of the unDRXed region [35]. The maximum value of the texture intensity of the DRXed region gradually increases with the increase of La content, which are respectively 5.3, 5.4 and 7.0, indicating that the grain orientation of the DRXed region gradually becomes more concentrated. Moreover, Figure 7 shows the distribution histogram of the basal slip Schmid factor of the extruded specimens. As the La content increases, the mean Schmid factor of the basal slip system $\{0001\}\langle 11\overset{-}{2}0\rangle$ slip system are 0.157, 0.182 and 0.164, respectively. When loading along the ED, the slip initiation of basal plane dislocations in Mg-3Al-1La-0.3Mn and Mg-3Al-5La-0.3Mn alloy requires greater stress than Mg-3Al-3La-0.3Mn alloy.

### 3.2. Thermal Conductivity of Mg-3Al-xLa-0.3Mn Alloys

Generally, the addition of alloying elements to Mg will inevitably deteriorate the periodic arrangement of Mg atoms, distort the crystal lattice of the Mg matrix, enhance the scattering of thermal conductive electrons and phonons, and reduce the mean free path of electrons and phonons [7], thereby reducing the thermal conductivity of the alloy [36,37,38]. With the increasing alloying element content, the thermal conductivity of the Mg alloy is significantly reduced [6,12,39]. Moreover, the addition of alloying elements gives rise to the generation of the second phases, which will also introduce new interfaces and/or cause lattice distortion of the Mg matrix, and accordingly, the thermal conductivity of the alloy would be reduced [40].

**Figure 8:**
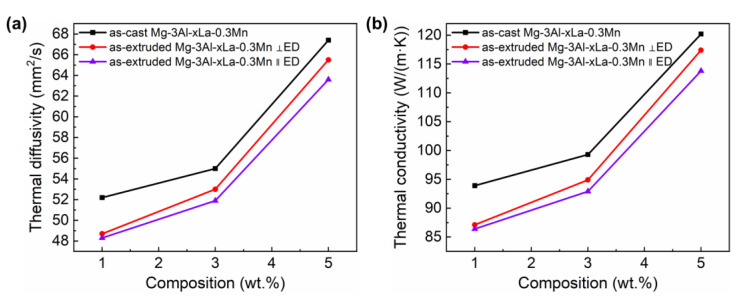
(**a**) Thermal diffusivity and (**b**) thermal conductivity of Mg-3Al-xLa-0.3Mn alloys.

Figure 8 shows the thermal conductivity and thermal diffusivity of the as-cast Mg-3Al-xLa-0.3Mn alloys. Obviously, the thermal conductivity of Mg-3Al-xLa-0.3Mn alloy is lower than that of pure Mg (156 W/(m·K)). Nevertheless, both thermal conductivity and thermal diffusivity increase with increasing La content. As shown in Table 3, the thermal conductivities of the as-cast samples Mg-3Al-1La-0.3Mn, Mg-3Al-3La-0.3Mn, Mg-3Al-5La-0.3Mn at room temperature are 93.9 W/(m·K), 99.3 W/(m·K), 120.2 W/(m·K), respectively. This is mainly because the added La atoms react with the Al in the Mg matrix to form the intermetallic compound Al_11_La_3_ phase, which reduces the concentration of solute atom Al in the matrix, then weakens the lattice distortion of the Mg matrix, inhibits the scattering of electrons/phonons, and therefore increases the thermal conductivity of the alloy [22]. As the amount of La increases, the content of the second phase increases significantly, which deteriorates the thermal conductivity. However, it was reported that the negative influence of alloying elements in the form of solute atoms in the matrix on thermal conductivity is an order of magnitude higher than that of alloying elements in the form of the second phase in the Mg matrix [23,41]. In the present work, the thermal conductivity of the as-cast alloy gradually increases with the La content increasing due to the combined impact of solute atoms and the second phase.

After extrusion, the average grain size is remarkably refined, the grain boundary volume fraction and dislocation density are significantly increased. Lots of grain boundaries and dense dislocations become the scattering sources that hinder the motion of electrons and phonons [7,42]. Meanwhile, the regularity of the Mg lattice arrangement is also greatly reduced, so the thermal conductivity of the extruded Mg-3Al-xLa-0.3Mn alloy is lower than that of the corresponding as-cast alloy. It should be noted that with the increase of La content, the difference of thermal conductivity between as-cast alloys and extruded alloys gradually decreases, which may be due to the fact that the reduction of thermal conductivity by dislocation density is higher than the number of grain boundaries. When the content of La is 1 wt.%, the recrystallization ratio of the extruded alloy is low, there are a lot of deformed grains with high-density dislocations, which has a strong hindering effect on the motion of electrons and phonons, so the thermal conductivity decreases the most, that is 6.8 W/(m·K). In contrast with as-extruded Mg-3Al-1La-0.3Mn alloy, the as-extruded Mg-3Al-3La-0.3Mn alloy and Mg-3Al-5La-0.3Mn alloy have higher recrystallization ratio and more grain boundaries but lower dislocation density in deformed grains. Therefore, the positive effect of decreasing dislocation density on thermal conductivity is greater than the negative effect of increasing grain boundary number. When the La contents are 3 wt.% and 5 wt.%, the reduction values of thermal conductivity are 4.4 W/(m·K) and 2.8 W/(m·K), respectively.

As the amount of La increases, the thermal conductivity and thermal diffusivity of the extruded alloy increase gradually. This is mainly due to the gradual increase of the second phase content of the alloy during extrusion, which consumes more solute atoms. In addition, the deformation structure region of the alloy decreases gradually, and the dislocation density gathered in these regions also decreases gradually. The study on the deformed Mg-Zn alloys done by Pan [43] showed that the dislocation density of the alloy increased sharply with the strain increasing, and the thermal conductivity of the alloy decreased gradually. However, after aging treatment, the dislocation density of the alloy decreases and the thermal conductivity of the corresponding alloy increases obviously. So, the decrease of dislocation density is beneficial to increase the thermal conductivity of the alloy. Consequently, the combined hindrance to the motion of electrons and phonons is weakened, and the thermal conductivity of the alloy increases gradually.

Besides, the thermal conductivity of extruded alloys is anisotropic, i.e., the thermal conductivity perpendicular to the ED is higher than that parallel to the ED. This is mainly on account of the distinct fiber texture features in the extruded alloys, whereby the basal planes of most grains are parallel to the ED, and the (0001) basal plane is the most closely packed. The resistance of electrons and phonons moving along the ED is greater, which reduces the mean free path of their movement so that the thermal conductivity parallel to the prismatic plane is bigger than that along the basal plane. This is consistent with the results of previous studies [7,42,44].

Furthermore, since the order of texture strength of extruded alloys is Mg-3Al-1La-0.3Mn > Mg-3Al-5La-0.3Mn > Mg-3Al-3La-0.3Mn, when considering the effect of texture strength upon the thermal conductivity of extruded alloys in different directions, the difference of thermal conductivity that perpendicular to and along the ED should be in the corresponding order for the extruded alloys with different La content. However, this is not the case. With the addition of La, the difference of thermal conductivity perpendicular to the ED and parallel to the ED gradually increases, which may be because the number and orientation of the second phase in the alloy have more influence on the thermal conductivity than the texture. With the increase of La composition, the second phase of the extruded alloy increases and is distributed in a strip along the ED. When heat conduction is carried out along the ED, the hindrance of the second phase particles to the movement of electrons and phonons is gradually enhanced. Therefore, the difference of thermal conductivity perpendicular to the ED and along the ED increases with the increase of La concentration.

### 3.3. Mechanical Properties of Mg-3Al-xLa-0.3Mn Alloys

**Figure 9:**
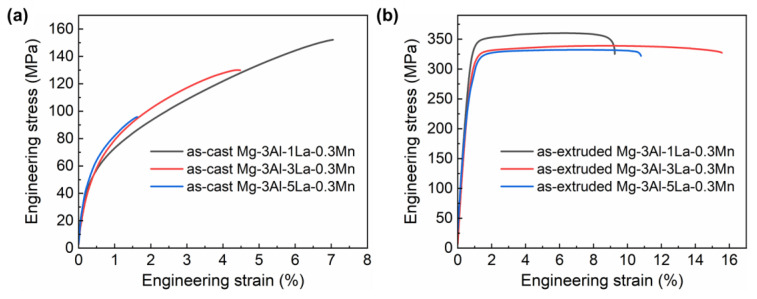
Tensile curves of as-cast (**a**) and as-extruded (**b**) Mg-3Al-xLa-0.3Mn alloys.

The tensile curves of Mg-3Al-xLa-0.3Mn alloys obtained at room temperature are shown in Figure 9. The detailed mechanical properties of the alloys are summarized in Table 4.

For the as-cast Mg-3Al-xLa-0.3Mn alloys, as the amount of La increases, the yield strength of the alloys increases gradually, while the tensile strength and elongation decrease gradually. This is because the grain size reduces and the volume fraction of the second phase increases and gradually changes from dispersion distribution to network distribution with the La content increasing. According to the Hall–Petch formula, the yield strength should gradually increase. The yield strength of the second phase is greater than the Mg matrix and can hinder the movement of dislocations. Therefore, the yield strength of the alloys increases with the second phase increasing [45]. However, as a hard and brittle phase, the second phase gradually presents a continuous network distribution along the grain boundary as the amount of La increases, which is not coordinated with the deformation of the Mg matrix during the tensile process and has a stronger cleavage effect on the matrix, which easily leads to the concentration of stress and the initiation of crack at the interface. Therefore, the increase of the brittle second phase results in the decrease of the plasticity of the alloy, which indirectly leads to the decrease of tensile strength [46].

After extrusion, the strength and elongation of the alloys are prominently increased compared to the as-cast alloys, which is mainly because of the significant refinement of the grains, the fragmentation of the second phase, and the generation of numerous dislocations during the hot extrusion process. As the amount of La increases, the tensile strength and yield strength of the alloys gradually decrease, and the elongation first increases and then decreases, which is the result of the combined effect of the texture strengthening and second phase strengthening.

The texture is an important factor affecting the mechanical properties of the alloys. With the increase in La concentration, the fiber texture strength of the alloy basal plane decreases first and then increases. The deformed grains show the strong basal plane texture, while the DRXed grains show weak basal plane texture. As shown in Figure 6, when La content is 1 wt.%, the alloy has the lowest recrystallization ratio and many deformed grains with strong basal plane texture. Its strength value is 17.2 multiples of random distribution (MRD). When the tensile test is performed along the ED, dislocations are difficult to slip along the basal plane, and the strengthening effect is significant. However, strain localization is easy to occur due to the poor strain coordination ability of coarse deformed grains. Once the plastic instability occurs, it is easy to fracture, so the elongation is low. In addition, a large amount of deformed grains have high-density dislocations and other substructures, dislocation entanglement occurs in the process of deformation, which hinders the initiation of the slip system, thus unfavorable to room temperature tensile plasticity. However, it can improve the yield strength of the alloy through obstructing dislocation movements. Therefore, the alloy shows the highest yield strength and tensile strength and the lowest elongation.

When La content increases to 3 wt.%, the proportion of recrystallized grains increases significantly, the orientation of DRXed grains is relatively random, and the average Schmid factor of basal plane slip increases. However, the percentage of deformed grains decreases and the basal plane texture strength decreases to 7.6 MRD, so the basal plane slip can be activated at relatively low stress, which reduces the yield strength of the alloy. The reduction of basal texture may ameliorate the plasticity of the alloy, because in the process of tensile test, the basal plane of some grains will deflect at a certain angle along the ED, making the basal plane change from hard orientation to soft orientation, which is conducive to plastic deformation and increase the elongation of the alloy, and the decrease of dislocation density will also reduce the strength of the alloy. As the La content reaches 5 wt.%, although the texture strength of the alloy increases to 9.2 MRD, the grain orientation of the extruded alloy changes. The (0001) basal planes of most deformed grains are parallel to the ED, while the orientation of most deformed grains in the extruded Mg-3Al-3La-0.3Mn alloy is $\left(2\overset{-}{1}\overset{-}{1}0\right)$‖ED. The critical shear stress of prismatic slip is much larger than basal slip around room temperature in Mg alloys. Therefore, the extruded Mg-3Al-5La-0.3Mn alloy requires relatively small stress to realize the actuating of the slip system, and the strength of the Mg-3Al-5La-0.3Mn alloy is less than that of the Mg-3Al-3La-0.3Mn alloy. However, as the amount of La increases, the volume fraction of the second phase in the extruded alloy increases. A large amount of broken intermetallic compounds can lead to stress concentration during the tensile deformation process and further lead to the generation of microcracks, which will reduce the elongation of the alloy. Therefore, the elongation of Mg-3Al-5La-0.3Mn alloy is lower than that of Mg-3Al-3La-0.3Mn alloy.

## 4. Conclusions

The microstructure, mechanical properties and thermal conductivity of as-cast and as-extruded Mg-3Al-xLa-0.3Mn (x = 1, 3 and 5 wt.%) alloys were studied. The conclusions are as follows:

- The thermal conductivity of the as-cast Mg-3Al-xLa-0.3Mn alloys increases gradually with the La content, which is due to the gradual increase of Al_11_La_3_ phase content and the decrease of solute Al in the Mg matrix.
- The yield strength of the as-cast alloys gradually increases with the increase of La content, while the tensile strength and elongation possess the opposite trend, which is attributed to grain refinement, the increase of second phase content and the change of distribution morphology.
- The thermal conductivity of the as-extruded alloys is lower than that of the as-cast ones due to the generation of defects such as a large amount of grain boundaries and dislocations. In addition, anisotropic thermal conductivity is generated because of the texture, i.e., the thermal conductivity perpendicular to the ED is higher than that parallel to the ED.
- After extrusion, the mechanical properties of the alloy are remarkably enhanced, while the yield strength and tensile strength of the as-extruded alloys gradually decrease with the La content increasing, and the elongation possesses a peak value with La content of 3 wt.%. This is owing to the combined effect of the texture strengthening and second phase strengthening.

## Figures and Tables

**Table 1:** Chemical composition of Mg-3Al-xLa-0.3Mn alloys.

Alloys	Composition (wt.%)			
	Al	La	Mn	Mg
Mg-3Al-1La-0.3Mn	2.73	0.99	0.29	Bal.
Mg-3Al-3La-0.3Mn	2.58	2.86	0.25	Bal.
Mg-3Al-5La-0.3Mn	2.85	5.07	0.30	Bal.

**Table 2:** Chemical composition of the phases marked in Figure 1d,e.

Positions	Chemical Compositions (at.%)				Phase
	Mg	Al	La	Mn	
A	44.26	33.91	5.32	16.51	Al_8_Mn_5_
B	92.42	6.73	0.77	0.08	Al_11_La_3_
C	92.13	6.71	1.02	0.13	Al_11_La_3_
D	95.69	4.21	0.02	0.08	α-Mg
E	83.26	13.82	2.74	0.17	Al_11_La_3_
F	82.47	10.36	7.06	0.11	Mg_12_La
G	86.08	10.56	3.24	0.13	Al_11_La_3_
H	99.22	0.62	0.02	0.14	α-Mg

**Table 3:** Thermal conductivity of Mg-3Al-xLa-0.3Mn alloys.

Alloys	As-Cast Thermal Conductivity (W/(m·K))	As-Extruded Thermal Conductivity (W/(m·K))	
		⊥ED	∥ED
Mg-3Al-1La-0.3Mn	93.9	87.1	86.4
Mg-3Al-3La-0.3Mn	99.3	94.9	92.9
Mg-3Al-5La-0.3Mn	120.2	117.4	113.8

**Table 4:** Mechanical properties of Mg-3Al-xLa-0.3Mn alloys.

Alloys	Yield Strength (MPa)	Tensile Strength (MPa)	Elongation to Failure (%)
as-cast Mg-3Al-1La-0.3Mn	57.0 ± 2.1	152.1 ± 6.7	7.1 ± 1.1
as-cast Mg-3Al-3La-0.3Mn	62.9 ± 3.2	130.1 ± 5.6	4.5 ± 0.5
as-cast Mg-3Al-5La-0.3Mn	63.8 ± 2.1	95.8 ± 6.2	1.6 ± 0.4
as-extruded Mg-3Al-1La-0.3Mn	326.2 ± 3.3	360.2 ± 6.9	9.2 ± 0.6
as-extruded Mg-3Al-3La-0.3Mn	309.1 ± 6.1	339.1 ± 6.5	15.6 ± 1.1
as-extruded Mg-3Al-5La-0.3Mn	271.0 ± 2.4	332.2 ± 5.1	10.8 ± 1.3

## Data Availability

The data presented in this study are available on request from the corresponding author.
